# Cassava Frogskin Disease: Current Knowledge on a Re-Emerging Disease in the Americas

**DOI:** 10.3390/plants11141841

**Published:** 2022-07-14

**Authors:** Juan Manuel Pardo, Elizabeth Alvarez, Luis Augusto Becerra Lopez-Lavalle, Cristian Olaya, Ana Maria Leiva, Wilmer Jose Cuellar

**Affiliations:** 1 Cassava Program, Crops for Nutrition and Health, International Center for Tropical Agriculture (CIAT), The Americas Hub, Km 17 Recta Cali-Palmira, Cali 763537, Colombia; j.m.pardo@cgiar.org (J.M.P.); e.alvarezca@outlook.com (E.A.); l.a.becerra@cgiar.org (L.A.B.L.-L.); a.m.leiva@cgiar.org (A.M.L.); 2 Department of Plant Pathology, University of California, Davis, CA 95616, USA; colaya@ucdavis.edu

**Keywords:** cassava, root yield, cassava frogskin disease, storage root, sugar content

## Abstract

Cassava frogskin disease (CFSD) is a graft-transmissible disease of cassava reported for the first time in the 1970s, in Colombia. The disease is characterized by the formation of longitudinal lip-like fissures on the peel of the cassava storage roots and a progressive reduction in fresh weight and starch content. Since its first report, different pathogens have been identified in CFSD-affected plants and improved sequencing technologies have unraveled complex mixed infections building up in plants with severe root symptoms. The re-emergence of the disease in Colombia during 2019–2020 is again threatening the food security of low-income farmers and the growing local cassava starch industry. Here, we review some results obtained over several years of CFSD pathology research at CIAT, and provide insights on the biology of the disease coming from works on symptoms’ characterization, associated pathogens, means of transmission, carbohydrate accumulation, and management. We expect this work will contribute to a better understanding of the disease, which will reflect on lowering its impact in the Americas and minimize the risk of its spread elsewhere.

## 1. Introduction

**Figure 1:**
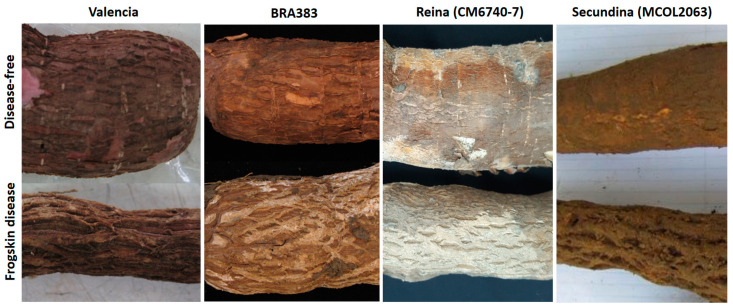
Root symptoms of CFSD as observed in four different field-infected cassava genotypes: Valencia, BRA383, Reina (CM6740-7), and Secundina (MCOL2063). Top pictures correspond to roots showing no symptoms of the disease. Bottom pictures show the characteristic root symptoms of CFSD.

Cassava frogskin disease (CFSD) was first described in 1971 from severely affected fields in the Department of Cauca, in southern Colombia, causing significant yield losses in cassava (*Manihot esculenta* Crantz) [1]. Cassava is originally from South America and is a key food security crop for millions of people in the tropics and is an important source of industrial starch worldwide [2]. CFSD affects the roots of the cassava plant, causing characteristic longitudinal lip-like fissures, with the root peel presenting a thick cork-like appearance (Figure 1). Most importantly, the storage root yield, in terms of fresh weight or starch content, is significantly reduced over additional crop cycles [3] (Supplementary Figure S1). Except for a few indicator genotypes (see below), the aboveground parts of the plant, such as the stems and leaves, do not show symptoms of disease [3]. The latter is a major factor in the dissemination of CFSD, as pieces of stems (stakes) are used as propagation material for the next crop cycles.

**Figure 2:**
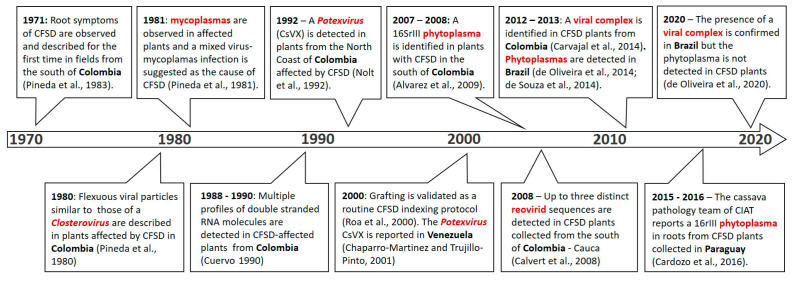
A timeline of the main results on the search for the causal agent of CFSD. Pineda et al., 1983 [1], Pineda et al., 1980 [7]; Pineda et al., 1981 [8]; Cuervo 1990 [9]; Nolt et al., 1992 [10]; Roa et al., 2000 [11]; Chaparro-Martinez and Trujillo-Pinto, 2001 [12]; Alvarez et al., 2009 [13]; Calvert et al., 2008 [14]; Carvajal et al., 2014 [15]; de Oliveira et al., 2014 [16]; de Souza et al., 2014 [17]; Cardozo et al., 2016 [18]; de Oliveira et al., 2020 [19].

In Colombia, CFSD is distributed throughout the main cassava-producing regions, where cassava is still widely cultivated under traditional practices [2,3]. The characteristic root symptoms of CFSD had been officially reported in Venezuela, Brazil, and Paraguay (Figure 2), and to the best of our knowledge, the disease does not occur in Africa or Asia [4]. Nevertheless, the recent events of transboundary movement of cassava diseases such as Cassava Mosaic Disease (CMD), spreading into Southeast Asia [5], and Cassava Common Mosaic Disease (CCMD), once limited to the Americas and now emerging in continental China [6], are warning signs that underline the potential of CFSD as a global threat to cassava production.

Since its emergence in Colombia, CFSD has been identified by observation of its characteristic root symptoms (Figure 1). However, reproducing such symptoms under experimental controlled conditions is not straightforward. A disease-free in vitro cassava plant will not produce fully developed storage roots during the first crop cycle, under greenhouse conditions. Except for susceptible plants used as indicators, which can show symptoms of the disease during the first 8–12 months under strong inoculum pressure, for most cassava genotypes it can take 2 or 3 crop cycles for CFSD root symptoms to develop (Table 1; Supplementary Figure S1). This characteristic may be related to the nature of the inoculum, which to date includes several pathogens occurring in mixed infections [7,8,9,10,11,12,13,14,15,16,17,18,19] (Figure 2).

Some susceptible genotypes showing severe CFSD root symptoms, such as ‘Secun-dina’ (MCOL2063), also express leaf symptoms (including leaf deformation, mosaic, yellowing), an observation that led to the development of a CFSD biological indexation test using grafting [9]. Leaf symptoms in these indicator genotypes develop 3–4 weeks after grafting on rootstocks from plants affected by CFSD. This test, although effective, readily showed that the kind and severity of leaf symptoms induced by CFSD-affected rootstocks vary, unraveling the occurrence of distinct mixed pathogen infections in affected plants [15]. After molecular characterization and detection of several viruses found in these plants (Table 2), grafting tests showed that in single infections, only cassava common mosaic virus (CsCMV) and cassava torrado-like virus (CsTLV) induce leaf symptoms in the indicator gneotypes Secundina and Reina [20,21]. A recent report from Brazil confirmed the occurrence of these mixed infections in a field collection of plants with root symptoms of CFSD [19]. Nowadays, grafting tests using indicator plants could still be used to identify the presence of mixed infections, but it is not recommended for general virus indexing. Additional protocols have been validated to detect these pathogens in cassava without the need for grafting [20,21,22,23,24] (Table 2).

Several aspects of CFSD biology are anecdotic or remain unknown. The objective of this review is therefore to update the current knowledge on CFSD gathered through different works carried out at CIAT over several field and laboratory trials. Among these, we include: an analysis of the changes occurring in carbohydrate accumulation in affected cassava roots, mode of transmission, management, and pathogens found in CFSD-affected plants.

## 2. Symptoms, Transmission, and Geographical Distribution

CFSD is not associated with symptoms in leaves or stems in most cassava genotypes. Therefore, the disease is commonly propagated via the distribution of stem cuttings (stakes), from plants that can actually have severely affected roots [25]. The characteristic root symptoms that give the disease its name occur in the storage roots of the cassava plant. These appear as longitudinal lip-like fissures, with the root peel presenting a thick cork-like appearance reminiscent of the rugose skin of some animals, hence the name frogskin or jacaré/caiman (Figure 1). When the stakes of an affected plant are used over additional crop cycles, the severity of the symptoms gradually increases, limiting the development of the storage roots, reducing root yield, and in the most severe cases, completely impeding starch accumulation.

After its first report in the south of Colombia, in the 1970s, the characteristic root symptoms of the disease have been reported in all the main Colombian regions where cassava is grown [1,11,15,26]. Here, it is worth mentioning that during surveys in the North Coast of Colombia in the early 1990s, root symptoms of CFSD were also associated with leaf mosaic symptoms, probably due to Secundina being at the time a popular genotype grown in this region. The leaf symptoms were then known as Caribbean Mosaic [11]. Observations such as these prompted the use of Secundina as an indicator plant for CFSD, as described in the previous section. Nevertheless, extensive field inspections in Colombia, Paraguay, and Brazil consistently show that the symptoms developed in Secundina are an exception, and that most genotypes that show root symptoms of CFSD do not develop leaf or stem symptoms [18,19,27]. In Venezuela, most genotypes with root symptoms of CFSD did not show symptoms in the aerial part of the plant, unless they were grafted to Secundina [13].

**Figure 3:**
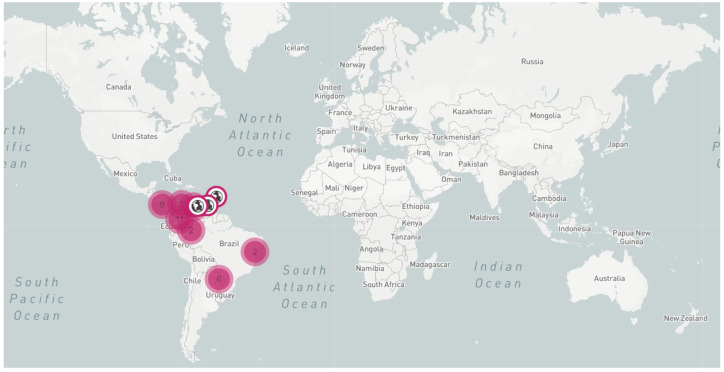
Map showing the current geographical distribution of CFSD according to a global literature search using Google Scholar, using search terms ‘mandioca couro de sapo’, ‘yuca cuero de sapo’, and ‘cassava frogskin’. Information from internal CIAT annual reports is included. The interactive map and links to the historical reports are available at: https://pestdisplace.org/embed/news/map/disease/5 [28]. The information was last accessed on 20 April 2022.

A thorough review of the literature and official country reports [28] show that CFSD geographical distribution is still limited to the Americas (Figure 3), and that the described root symptoms are clearly distinct from those related to other diseases affecting cassava roots, e.g., Cassava Brown Streak Disease [29]. Nevertheless, due to the asymptomatic nature of CFSD-affected stakes, and the absence of strong phytosanitary regulations and seed certification protocols, CFSD is a potential threat to cassava cultivation in other regions. Protocols for CFSD early identification and diagnostics are urgently needed.

Although there is a fair amount of literature about this disease (mostly in Spanish), field visits evidence the still existing lack of information surrounding CFSD biology among smallholder farmers and other stakeholders. For example, although it is clear that stems from affected plants are a source of the disease, a still general field practice is to collect cassava stems and pile them up before harvesting the roots (pre-harvest pruning). As a result, stems from affected plants are neither identified nor discarded [25]. In other cases, farmers unfamiliar with the disease point out the soil as a source of inoculum and thus avoid planting cassava in plots where they observed high incidences, but re-use their contaminated stems, bringing the disease to new areas. Although soil transmission is plausible, assays where disease-free plants were grown in soil collected from fields with a high incidence of CFSD indicated that soil is not a source of inoculum. Furthermore, CFSD transmission studies, carried out at CIAT’s field station in Santander de Quilichao (Department of Cauca, Colombia), evaluated the expression of root symptoms in the CFSD-tolerant genotype HMC-1, under high disease pressure, using a severity scale of 1 to 5, where 1 = asymptomatic (none of the roots showed symptoms) and 5 = severe (high density of lip rows resembling a honeycomb-like structure covering the whole root, reduced storage root thickness, and numerous fibrous roots). In a three-year experiment [30], two groups of disease-free plants were established one next to the other, in blocks surrounded by cassava fields with high incidences of CFSD. One group was protected inside a muslin screen house (insect-proof). All those originally disease-free plants, growing in the open field, developed typical root symptoms of CFSD, at higher frequencies over the three years of the experiment. At the same time, all disease-free plants that were grown under insect-proof conditions (but exposed to the same soil as those in the open field) remained disease-free over the course of the experiment (Table 1). We reasoned that if soil was a source of inoculum, both groups of plants would have been equally affected by CFSD; nevertheless, disease-free plants grown in an insect-proof screen house remained symptomless throughout the three-year experiment (Table 1). If soil were not the source of the inoculum, infection of the disease-free plants in the open field would require the presence of aerial vectors. In fact, a fraction of disease-free plants growing in the open field and treated with insecticide showed lower percentages of affected root during the three years of the experiment. These results are summarized in Table 1.

Olaya [31] evaluated botanical seeds (*n* = 222) obtained from plants of the genotype CMC40 (COL1468), showing severe root symptoms of CFSD. The seeds were germinated in an insect-proof greenhouse, obtaining a percentage of germination of 74.3%. Out of 151 tested plants, none of them showed symptoms of the disease and all resulted negative (6 months after planting) to all the pathogens reported in CFSD-affected plants (see Section 4). In contrast, the parental plants were positive for several of the associated viruses and phytoplasma. Although we cannot directly discard the transmission of CFSD by botanical seeds, it seems highly inefficient, at least for the genotype evaluated. Moreover, as cassava is propagated by using stakes rather than by botanical seeds, a significant contribution of the latter to the dissemination of CFSD (and its associated pathogens) in the field is unlikely.

In contrast to botanical seed, transmission of CFSD by chip-bud grafting is highly efficient [23]. Chip-bud grafting from infected plants into indicator recipient genotypes such as Secundina or Reina (CM6740-7) is used in our laboratory as a routine method for virus transmission. When the CFSD inoculum derives from plants showing severe root symptoms, the recipient indicator plant could express leaf symptoms four weeks after grafting [21,31] and root symptoms after the first crop cycle (10–12 months), and the severity of the root symptoms increases overtime. As a positive control of CFSD inoculum, we maintain and use as a source an infected plant coded 5460-10, originally collected from the department of Cauca (south Colombia) and used as an inoculum in early screenings for CFSD resistance at CIAT. This inoculum contains a complex mixed infection of viruses and phytoplasma [15,31]. Together with the cassava indicator Reina, they have been used to reproduce the root symptoms of CFSD [21] (Supplementary Figure S1). Analysis of the effect of each individual associated pathogen requires its isolation in single infection, reinoculation, and recording of the development of root symptoms, which so far has not been possible [20]. An alternative to isolate each pathogen from a mixed infection has been to screen cassava plants looking for single-pathogen infections and follow-up the development of symptoms in leaves and roots (see Section 4).

## 3. CFSD Affects the Yield and Sugar Content of Roots

Cassava is a food security crop, valued mainly by its storage roots that accumulate a high content of sugars in the form of starch [2]. Changes in the metabolism of these sugars (and related organic acids) are good indicators of the health status and quality of the roots [32]. Although our understanding of the mechanisms promoting the accumulation of starch in the roots of cassava is still limited, it is clear that one of the effects of CFSD is the reduction of starch accumulation and yield of cassava roots, altering the rates of sugars and organic acids [33]. At the same time, several reports indicate that carbohydrate metabolism can be altered during pathogen infections [34,35,36].

**Figure 4:**
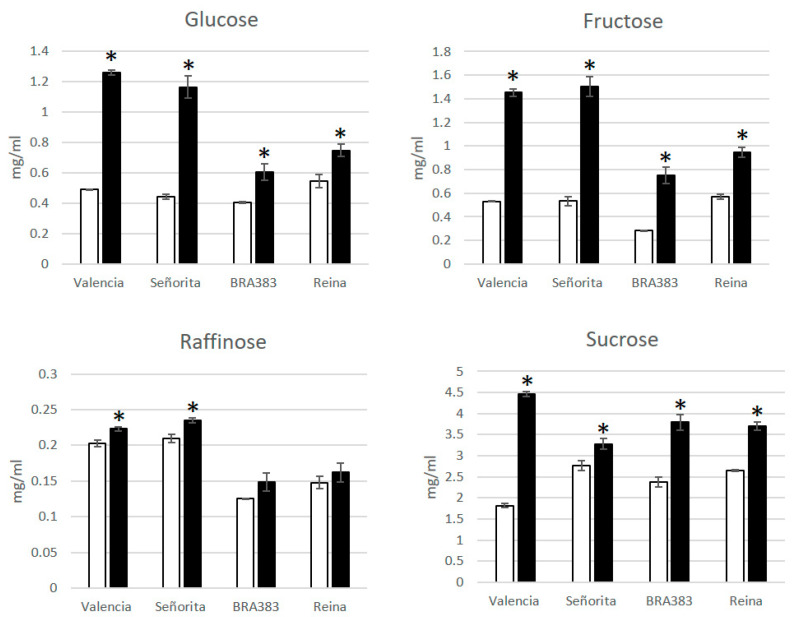
Sugar content in four different genotypes from Costa Rica (Valencia and Señorita) and Colombia (Bra383 and Reina). Dry matter was expressed as the percentage of dry weight relative to fresh weight. Sugar (and organic acids) analysis was carried out from a 500 mg flour sample. Two determinations were carried out with each flour sample. Sugars were analyzed using HPLC (Agilent Technologies 1200 series, Waldbronn, Germany). Samples were separated isocratically at 0.6 mL/min and at 30 °C and retention times and standard curves were prepared for glucose (Sigma-Aldrich, St. Louis, MO, USA, G7528), fructose (Sigma-Aldrich F2543), and saccharose (Sigma-Aldrich; ≥99.5%; S7903). *p*-values < 0.05 (*) were considered as significant. Error bars are the mean +/− SEM (standard error of the mean).

To better understand the effect of the disease in cassava starch accumulation, Alvarez et al. [33] collected roots of symptomatic and asymptomatic plants from fields in Costa Rica and analyzed the sugar content in flour from roots (four biological replications per genotype and two genotypes). The parameters included dry matter content and concentrations of reducing sugars, total sugars, and organic acids, using HPLC. A similar experiment was conducted previously with two other genotypes in Colombia (unpublished). When the data were disaggregated by metabolites, results showed that the roots of four CFSD-affected genotypes (Valencia and Señorita in Costa Rica and Bra383 and Reina in Colombia) had a significantly higher concentration of glucose, fructose, and sucrose (Figure 4). These three mutually related sugars are all associated with starch metabolism, and the changes observed are most likely related to a mobilization of starch reserves as part of the activation of physiological pathways to fight an infection [37,38]. This would also result in lower levels of starch content in storage roots of infected plants. In fact, dry matter in CFSD-affected roots diminished by 5.73% and 10.88% in Valencia and Señorita, respectively [33], and by 4.76% and 5.84% in BRA383 and Reina. A reduced dry matter accumulation is related to a reduced starch yield and has already been reported in relation to virus infections in cassava by other groups [39,40]. Among the organic acids, malic and succinic acids showed a significant increase in CFSD-affected roots in the four genotypes evaluated. Of the two, malic acid was found to be the highest in concentration in diseased roots, correlating with the amounts of reducing and total sugars, particularly fructose (R2 = 95%) (Figure S1). Globally, cassava roots are the second most important source of starch after maize [41]. The comparative effect of distinct pathogens, specifically on root starch content in different cassava genotypes, requires further study.

## 4. CFSD-Associated Pathogens and Molecular Diagnostics

The graft-indexing protocol using Secundina described above consistently reproduced leaf mosaic symptoms when the rootstocks were from plants with clear root symptoms of CFSD [15,31]. Another genotype, named Reina, is also routinely used as an indicator of the disease. In the absence of alternative diagnostic methods, grafting into indicator plants, a test that on average takes 4–5 weeks for leaf symptoms to develop, was readily implemented as an indexing method for the cassava germplasm collection at CIAT [9]. Nevertheless, further tests that started in 2012 demonstrated that this method was not efficient in detecting single infection of several cassava viruses [15,31]. Furthermore, cassava requires at least one crop cycle to produce storage roots, and therefore the whole process may require at least two years, as the roots formed during the first harvest (under screen house conditions) do not develop uniformly. As the Secundina indexing protocol could not include rooting of the cassava plants to be validated for CFSD root symptoms in the context of a large-scale screening (required for routine germplasm indexing, resistance trials, or disease surveillance activities), alternative indexing methods had to be developed.

As shown in Figure 2, since its first report, CFSD has been associated with different pathogens. It is noteworthy that all pathogens found in CFSD-affected plants have been reported only in the Americas, coinciding with the occurrence of the distinct root symptoms of CFSD reported only in this region (Figure 3). For almost two decades (1971 to 1989), pathogens found in CFSD samples were identified by describing the shape of their structures under the electron microscope or the symptoms observed in the affected plants [1,7,8,9,10,11]. These early analyses already pointed to the presence of several pathogens present in CFSD-affected plants, most likely in mixed infections. It was in 2008 that a reovirid and a phytoplasma were genetically identified in CFSD-affected plants, and the first molecular diagnostic tests were developed [12,14].

**Figure 5:**
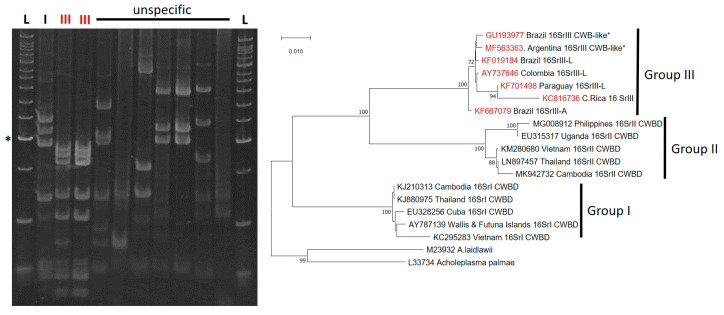
Phytoplasmas detected in CFSD-affected plants belong to the 16Sr ribosomal group III (in red). Restriction analysis (**left**) of the 16S RNA region amplified by nested PCR. Phylogenetic analysis of the 16Sr sequences of phytoplasmas isolated from cassava (**right**) in the Americas (in red) and Southeast Asia. L = Ladder. CWBD = cassava witches’ broom disease. L = DNA Ladder (Bioline, USA). The asterisks indicates the 300 bp band.

Phytoplasmas: Phytoplasmas are a class of bacteria that lack a cell wall and are obligate parasites of their hosts, where they can trigger distinct symptoms, such as the proliferation of stems and the conversion of flowers into leaves, known as phyllody [42,43]. They can be classified based on the phylogenetic relationship and Restriction Fragment Length Polymorphism (RFLP) analysis of their 16S rRNA into ribosomal groups and subgroups [44]. Cassava-infecting phytoplasmas from the Americas belong to ribosomal group 16Sr III, separated from cassava phytoplasmas found in Southeast Asia related to ribosomal groups 16Sr I and II and associated with phyllody symptoms [45] (Figure 5). Phytoplasmas of the 16Sr III group have been detected in CFSD-affected plants in Colombia, Paraguay, Costa Rica, and Brazil [16,17,18,24,27]. As mentioned above, determining the effect of a pathogen in the development of a disease traditionally requires its isolation in pure culture (which is not possible with phytoplasmas), and one to two years to observe root symptoms in cassava. Alvarez et al. [12] were able to isolate and inoculate a phytoplasma using dodder plants; nevertheless, the assay could not be conducted until harvest and therefore no root symptoms could be reported in single phytoplasma infection. In our laboratory, phytoplasma detection is not as straightforward [24], as it requires nested PCR protocols to account for the generic nature of the primers used, the low titers of the pathogen, and its unequal distribution in plants [46,47]. For example, 16Sr III phytoplasma have been reported to be associated with CFSD in Minas Gerais, Brazil [17], but another study indexing CFSD in a collection of field plants in Bahia reports the presence of mixed virus infections but could not detect phytoplasma [19].

Oryzavirus: Viruses have long been associated with root diseases in cassava. The best example is that of the cassava brown streak virus (CBSV) causing necrotic symptoms in affected roots [29] and the recent reports from Venturini et al. [39] and Collavino et al. [40], showing a reduction of up to 30% in root yield associated with the alphaflexivirid CsCMV. Similarly, more than 10 different species of geminivirids are reported to cause dramatic root yield reductions in Africa and Asia [4], indicating the potential of viruses to cause dramatic reductions in root yield. Among the best characterized viruses in CFSD-affected plants, there is a reovirid named cassava frogskin-associated virus (CsFSaV; Genus *Oryzavirus*; Fam. *Reoviridae*) [14,31]. Although the exact role of reovirids in the formation of root symptoms remains uncertain, the results from surveys by different groups have consistently found them in all fields were CFSD is reported [17,19,31,48]. Neither CFSD nor cassava-infecting reovirids have been reported in Africa or Asia.

**Figure 6:**
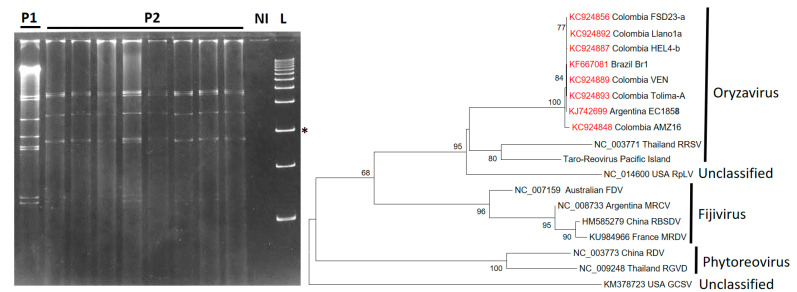
Distinct patterns of dsRNA detected in cassava plants affected by CSFD. Cuervo reported at least two dsRNA patterns in plants with CFSD [10]. Both patterns show 10 dsRNA bands. Left: Patterns P1 and P2 can be detected in samples from the Amazon region of Colombia. P2 is more common in samples from other regions (photo kindly provided by M. Cuervo). NI = Not infected control. L = 1 kb ladder. The asterisks indicates the 2 kb band. Right: Phylogenetic analysis of reovirids infecting plants based on the amino acid sequence of the RdRp domain. RRSV = Rice ragged stunt virus (NC_003771); RpLV = Raspberry latent virus (NC_014600); FDS = Fiji disease virus (NC 007159); MRCV = Mal de Rio Cuarto virus (NC 008733); RBSDV = Rice black-streaked dwarf virus (HM585279); MRDV = Maize rough dwarf virus (KU984966); RDV = Rice dwarf virus (NC 003773); RGVD = Rice gall dwarf virus (NC_009248); GCSV = Grapevine Cabernet Sauvignon reovirus (KM378723).

The family *Reoviridae* is the most diverse of the double-stranded RNA (dsRNA) virus families, containing species that infect a wide range of hosts including mammals, birds, fish, insects, fungi, and plants. The genomes of these viruses are segmented (9–12 segments) and thus likely to re-assort, giving rise to variants (strains) whenever mixed infections occur [49]. Out of 15 genera, reovirids of genera *Phytoreovirus*, *Fijivirus*, and *Oryzavirus* can infect plants. The reovirid found in cassava with CFSD symptoms is named cassava frogskin-associated virus (CsFSaV), and it presents two different dsRNA patterns, as observed by eletrophoresis [14] (Figure 6). CsFSaV groups within the *Oryzavirus* genus share an amino acid sequence similarity of 57% for s4 (replicase) and 45% for s3 (coat protein) to Rice ragged stunt virus (RRSV), the type member of genus *Oryzavirus*. All predicted protein sequences from CsFSaV are closely related to their homologous proteins in RRSV. Phylogenetic analysis groups CsFSaV with RRSV and Raspberry latent virus (RpLV), which have also been found to be related to reovirids isolated from plant-feeding insects [31,50,51] (Figure 6).

Applying a new RT-PCR protocol to samples from grafting tests, we uncovered that rootstocks coming from in vitro plants positive only for CsFSaV did not develop leaf symptoms in the indicator plant [15]. Moreover, in single infections, only CsCMV and CsTLV induced leaf symptoms [20,21]. In contrast, all field-collected CFSD-affected rootstocks, presenting CsFSaV in mixed infections with other viruses, induced mild to severe leaf mosaics and leaf deformation symptoms in Secundina [15,31]. These results altogether allowed us to form two conclusions: (1) ‘Secundina’ is not an appropriate indicator plant for CsFSaV, CsPLV, CsNAV, or phytoplasma in single infections, and (2) additional pathogens in mixed infections with CsFSaV or phytoplasma may contribute to the observed leaf symptoms and possibly to root symptoms of CFSD.

Other pathogens: Since the first diagnostic works aiming to identify the causal agent of CFSD, different viral particles and bacteria-like agents have been observed in samples collected in Colombia and elsewhere (Figure 2). By applying a technology known as high-throughput sequencing of small interfering RNA to cassava samples with CFSD, and then using RT-PCR to confirm the presence of the virus contigs assembled, mixed virus infections were readily confirmed in Colombia [15,20,31] and then in Brazil [19]. These works confirmed the occurrence of at least three new viruses infecting cassava in the Americas, a polerovirus, a potexvirus, and a torradovirus, in different mixed infections with CsFSaV and phytoplasma. New sequencing technologies have been powerful tools to detect and identify viruses whose virions are difficult to isolate or purify, especially in clonally propagated crops such as sweet potato, potato, and cassava, where several pathogens can build up over successive crop cycles [52,53,54]. Nevertheless, we would like to highlight that the discovery of additional pathogens in CFSD-affected samples does not indicate that all of these can cause CFSD, even more when some of them can induce distinct symptoms in single infections, such as CsTLV [20]. More studies are needed to unravel the role of each pathogen (and their combination) in the etiology of CFSD.

As mentioned above, isolation by mechanical inoculation is not possible for most of these pathogens, and its common occurrence in mixed infections makes it harder to evaluate its individual or combined effect on cassava. Recently, Leiva et al. [26] reported the occurrence of CsTLV in mixed infections with phytoplasma in fields with a high incidence of CFSD in the Department of Casanare (eastern Colombia). Several years of experience working with these pathogens have validated mechanical isolation in *Nicotiana benthamiana* plants, only for CsCMV and CsVX, which are not associated with root symptoms of CFSD. We have implemented standardized field data collection and nucleic acid extraction protocols [5,22] for cassava pathogen detection and characterization (Table 2), which complement the chip-bud grafting [23] and greenhouse experiments described above. The availability of a novel virus vector that can be mechanically transmitted to cassava [55] could be helpful to study the role of different viruses (via RNA silencing) or viral genes (via transient expression) in the development of CFSD.

## 5. Genetics of CFSD Resistance and Breeding

As a response to increasing CFSD incidences, cassava farmers undertake visual inspection, in the field, to identify those cassava plants showing mild CFSD symptoms in the roots and manually eliminate the stems of those cassava plant affected. This method has been called “positive selection” by Ceballos and Hershey [25]. Although the method is effective to a large extent, it does not discriminate for the presence of any of the associated pathogens in visually asymptomatic cassava plants that are likely to build up infections, thus perpetuating the problem.

The detection of associated pathogens and/or the causal agents of CFSD can be addressed by molecular diagnosis, but it is logistically difficult and increasingly expensive to implement it at scale [12,14,15]. Alternatively, a thermotherapy-based control method has been put forward by using a polycarbonate chamber to create a field-based facility to eradicate the viruses and phytoplasma responsible for the root symptoms and rapidly increasing the production of clean planting materials [56]. Recently, an in vitro-based technique has been proposed as the most efficient method to eradicate viruses in cassava tissues by combining thermotherapy with shoot tip culture, chemotherapy, micro-grafting, or shoot tip cryotherapy [57]. Nevertheless, all these approaches are labor-intensive and resource demanding; hence, an alternative approach is needed to deliver a cost-effective solution to resource-poor cassava farmers.

The most cost-effective solution is introgressing CFSD resistance into advance cassava breeding materials, but finding reliable sources of resistance has been elusive [25]. Since the earliest reports of CFSD by CIAT, it has been difficult to identify both resistant and susceptible cassava genotypes that can be confidently placed under these two categories [58,59]. Alvarez et al. [60] confidently identified three CFSD-resistant (PER183, HMC-1, and CM4574-7) and two CFSD-susceptible (NGA11 and COL1505) genotypes, where NGA11 is ITTA’s TMS60444. CIAT increased its efforts to screen the germplasm collection for CFSD resistance, identifying an initial set of 58 CFSD-resistant and 114 CFSD-susceptible cassava genotypes among its global cassava germplasm collection (unpublished).

## 6. Conclusions

On symptoms: Several hallmarks of the biology of the root symptoms of CFSD are listed above. In early experiments, large volumes of soil samples were collected from high disease pressure fields and taken to screen houses at CIAT, where the disease could not be reproduced using susceptible stakes as experimental material. Other sets of experiments, such as the ones described here, took clean planting material to regions with high incidences of CFSD, uncovering that protecting the stakes from insects was sufficient to maintain the plants free of the disease for years (Table 1). These experiments point to airborne vectors as the most likely candidates for the horizontal transmission of CFSD root symptoms. Interestingly, symptomatic roots showed a reduced dry matter content (equivalent to reduced starch yield; see also Supplementary Figure S1), that correlates with increasing concentrations of reducing sugars in symptomatic roots (Figure 4). It appears that an increased respiration inducing the conversion of starch to sugar in cassava roots could be a more general response to biotic and abiotic stress in this crop [61,62].

On the causal agent: Several pathogens have been detected in CFSD-affected plants, as reported in several works (Figure 2). Mixed pathogen infections in cassava are commonly detected in the Americas, and isolation of each of them in single infections using indicator plants such as *Nicotiana* has not yet been possible, except for some alphaflexivirids [21,23]; therefore, the evaluation of these pathogens in different combinations remains a challenge. Two of the most studied pathogens in single infections, phytoplasmas and reovirids, seem not to be able to cause the disease by themselves. Current sequencing methods have unraveled a wider diversity of pathogens, at genus and family levels, infecting cassava in the Americas, occurring in mixed infections in plants with severe root symptoms of CFSD [15,16,19]. Nevertheless, large-scale statistical studies to identify the pathogens most closely associated with root symptoms of CFSD are still lacking. The potential of different vectors involved in the transmission of this diversity of pathogens also needs to be further considered.

On management: At the farm level, CFSD can be efficiently managed by the rigorous selection of propagation material (stakes from healthy plants) at every crop cycle [63], which has a direct effect on the building up of pathogens in the infected plants. Given the emerging characteristics of the disease, a model to address the study of CFSD could be that of ‘seed degeneration’, defined as the gradual decline in the quality of planting material over repeated cropping cycles. This decline may occur due to agronomic and abiotic factors or disease (by accumulation in titers of a pathogen or building up different pathogens over successive crop cycles) [64]. Although studies addressing the effect that distinct mixed infections can have on cassava are scarce, tools for specific detection of a list of pathogens reported in CFSD-affected plants are readily available. In Table 2, we list those used to screen for cassava pathogens in CFSD surveys carried out at CIAT, and they can be used to understand the relationship between yield reduction, CFSD root symptom severity, and the accumulation of the different pathogens (and their strains) over several crop cycles. Prevention of pathogen build up can be achieved by the periodical removal of infected plants and replacement using stakes coming from fields free of CFSD. This will require maintaining an area destined specifically to the production of disease-free quality stakes, which could guarantee a self-sufficient and profitable seed production system for farmers over the long term [25].

On breeding: New sources of CFSD resistance in cassava opened up the opportunity to transfer it into Sub-Saharan Africa, where it is not yet present. The co-occurrence of both CFSD and cassava brown strike disease (CBSD) in Africa would create a significant food crisis in the continent, making 800 million people food-insecure. Any future efforts to transfer CIAT sources of CFSD resistance into African cassava improved germplasm will require deploying genomic-based tools to guide modern breeding approaches. Marker-assisted selection, genomic selection, genetic transformation, and gene editing should be combined with the development of advance breeding populations (inbred cassava), allowing the crop’s breeder to exploit heterosis [59]. Furthermore, cassava geneticists and breeders should aim today at redesigning cassava so that it can be planted through botanical seeds, minimizing the spread of pathogens through stem cuttings.

CFSD is one major disease of cassava in the Americas, and potentially elsewhere [4,65]. Protocols for the rapid identification of symptoms and associated pathogens would support the early detection of CFSD-contaminated material and the evaluation of cleaning protocols applied to in vitro collections or field material, which so far are based on root symptom identification only [56]. As additional information on the biology and epidemiology of CFSD is integrated into breeding for resistance programs, the efficiency of current CFSD management practices can be improved and extended for the greater benefit of smallholder farmers in the tropics [25,59,60].

## Figures and Tables

**Table 1:** Three-year evaluation of CFSD root symptoms’ development under different conditions. Stakes from CFSD-affected plants came from the municipality of Jamundi, Valle del Cauca, and the disease-free stakes from the municipality of Montenegro, Quindío, where CFSD had not been reported before. Field trials were carried out in Santander de Quilichao (Department of Cauca, Colombia), under a high CFSD pressure. Fumigations were performed every 15 days using a mix of Malathion 1 mL/L, Thiamethoxam: 0.5 g/L, and Lambda-cyhalothrin: 2 mL/L. Each treatment used three repetitions of twelve plants each and a split plot experimental design. To record symptoms’ development per plant, one stake per plant was used for the next season. All stakes were treated with insecticide prior to planting (immersion in 2 mL/L of 0, 0-diethyl-S-methyl carbamoyl methyl phosphorodithioate).

Treatment	Seed type ^a^	Average Severity of CFSD Root Symptoms (% Infected Plants)		
		1st Crop Cycle (2004–2005) ^b^	2nd Crop Cycle (2005–2006) ^b^	3rd Crop Cycle (2006–2007) ^b^
Open field, no fumigation	CFSD	3.78 (77.3%)	3.72 (75.2%)	3.90 (79.3%)
Open field, no fumigation	Disease-free	1.00 (0.0%)	2.55 (34.3%)	2.60 (38.2%)
Open field + fumigation	CFSD	3.22 (57.7%)	3.77 (77.0%)	3.90 (81.0%)
Open field + fumigation	Disease-free	1.00 (0.0%)	1.60 (9.0%)	1.70 (15%)
Screen house + fumigation	CFSD	2.67 (38.5%)	2.93 (47.6%)	2.20 (33.3%)
Screen house + fumigation	Disease-free	1.00 (0.0%)	1.00 (0.0%)	1.00 (0.0%)

**^a^** Cassava stakes used as seed: CFSD = stakes obtained from diseased plants. Disease-free = Stakes obtained from disease-free cassava plants. **^b^** Average severity as observed in the roots of each treatment following the scale: 1 = asymptomatic, 2 = mild, 3 = moderate, 4 = moderate high, 5 = severe. The decimal indicates the average severity per plant using the most severe symptoms observed. The percentages shown indicate the number of diseased plants in each treatment.

**Table 2:** List of viruses and phytoplasma detected in plants showing root symptoms of CFSD and the PCR primer sequences used for generic diagnostics by the Cassava Crop Protection team at CIAT.

Family	Pathogen	Primers (5′ to 3′)	Test	Ref
*Reoviridae*	CsFSaV	CsFSaV-F: TGG CCG GGA GAA CAA TAA TA CsFSaV-R: GCG AAG TAA GTT CCG TCG TT	RT-PCR	[14]
*Secoviridae*	CsTLV	CsTLV-1F: GAC TCA ATG AAG GAG GAG GAT AGA CsTLV-1R: ACC AGA GCT TGT CCT AAT AGC AAC	RT-PCR	[15,20]
*Luteoviridae*	CsPLV	CsPLV-F2: TTG CAT TCA AAG ATC AGT TCT CTC CsPLV-R3: TGG TTG ACA GCT GTT TCA GAG G	RT-PCR	
*Alphaflexiviridae*	CsVX	CsVX-RdRp-F1: GCR TTG ACC AGG CAG TCA CCW GAC CsVX-RdRp-R1: TAG CCC TCT ATC ACG TCC TCA	RT-PCR	[21,23]
	CsCMV	CsCMV_3269F: GAG GCT CTT CTC TGG GAA AC CsCMV_3896R: CTT GAG TCC AGT TTG ATG TC	RT-PCR	
	CsNAV	CsNAV-RdRp-F: TGA GAG CAA TYT RAA GGA AA CsNAV-RdRp-R: GAT GAT ATC GTC AGG AAG AC	RT-PCR	
*Acholeplasmataceae*	Cassava frogskin Phytoplasma (16SrIII)	rpIII-PF: GAG AAG CAC AAG CAA TTT TGA TG rpIII-PR: CAG CGT TGG CAA CAG CAC Probe: FAM/ACC CCA AAA GCA GCT TCT CCA ATC G/BHQ	qPCR	[24]

## Data Availability

The data presented in this review are available from public documents and databases as indicated in the text. For some historical reports not available online, the documents can be requested from the corresponding author.

## Supplementary Materials

The following supporting information can be downloaded at: https://www.mdpi.com/article/10.3390/plants11141841/s1, Figure S1: Effect of CFSD on root yield of genotype CM6740-7 (‘Reina’). Disease-free plants were multiplied and inoculated with buds from infected plants showing severe root symptoms of CFSD or with buds from disease-free plants. A and B show the average root of one not infected and one infected plant, respectively after 3 crop cycles. C. Detail of the severe CFSD root symptoms in ‘Reina’. D. Yield losses of >80% can be recorded in this genotypes (average of 7 plants). Plants were maintained in a insect-proof greenhouse at CIAT throughout the 3-year experiment.
