# Supplementary material for: Cassava Frogskin Disease: Current Knowledge on a Re-Emerging Disease in the Americas
Source: Plants (Basel). 2022 Jul 14;11(14):1841. doi: 10.3390/plants11141841 (PMC9318364; doi:10.3390/plants11141841)
Supplement: Supplementary file 1 [file plants-11-01841-s001.zip › plants-1778961-SI.pdf]

Supplementary Figure 1

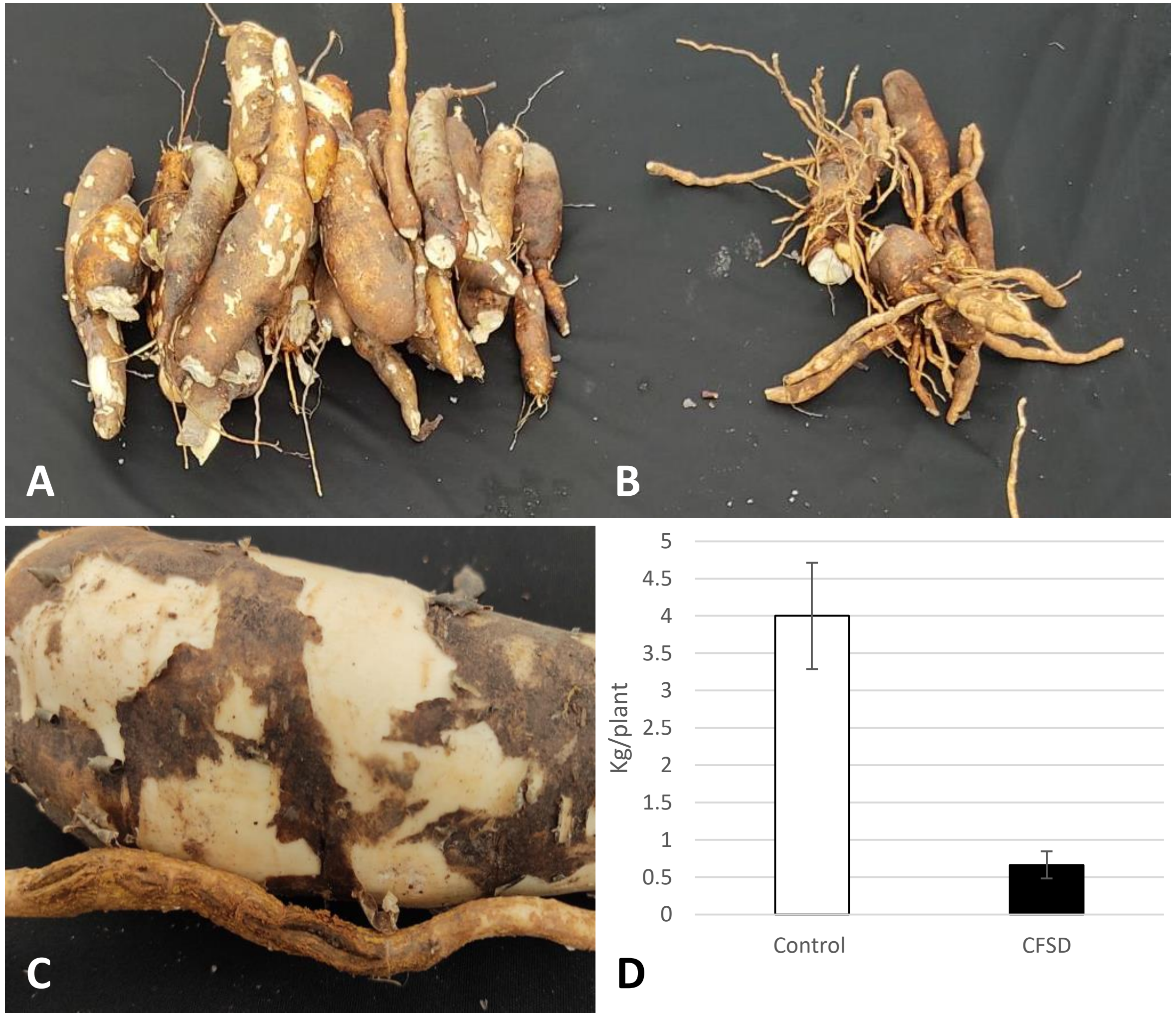

Figure S1. Effect of CFSD on root yield of genotype CM6740-7 (‘Reina’). Disease-free plants were multiplied and inoculated with buds from infected plants showing severe root symptoms of CFSD or with buds from disease-free plants. **A** and **B** show the average root of one not infected and one infected plant, respectively after 3 crop cycles. **C**. Detail of the severe CFSD root symptoms in ‘Reina’. **D**. Yield losses of > 80% an be recored in this genotypes (average of 7 plants). Plants were maintained in a insect-proof greenhouse at CIAT throughout the 3-year experiment.
